# Contextualized Contribution of Kindness to Favorable Goal- and Circumstantial-Driven Neuropsychological Regulation

**DOI:** 10.3389/fpsyg.2017.01643

**Published:** 2017-09-26

**Authors:** Nayara Mota, Elenilda Chaves, Marina Antunes, Rudi Borges, Andressa Paiva, Vanessa Santos

**Affiliations:** Department of Fundamentals of Psychology, Rio de Janeiro State University, Rio de Janeiro, Brazil

**Keywords:** kindness, self-regulation, executive functions, principles, cognition, contextual models

## Abstract

Kindness involves care and non-judgmental understanding toward someone. As a prosocial inclination, kindness would increase the possibility of favorable interaction with the environment, with a successful adjustment of one's response in novel or challenging circumstances, taking into account rules or goals. This adjustment ability is commonly referred to as executive functions, dependent on the prefrontal and parietal functioning, still under development during late adolescence. This study aimed to investigate if kindness would relate with the executive functions. If so, it would correlate more with measures of self-regulation, mainly dependent on the medial prefrontal corticosubcortical circuits. Also, among self-regulating processes, kindness would be more associated with autonomic responses—choices guided by one's understanding/intention - than with adaptive responses—changes on one's choices triggered by unfavorable circumstances. A sample of 46 (31 female; 18 to 21 years-old) healthy college students from the University of the State of Rio de Janeiro attended a clinical interview and a comprehensive neuropsychological assessment. Kindness was measured by the Compassion Scale subscore. Generalized non-linear models for each neuropsychological variable were executed on R, followed by an estimation of weighted parameters for each factor. Significant models which included kindness (weighted parameter Pc > 74) and all of their psychosocial or sociodemographic factors on their maximum expression (Pc > 74) were identified. In a contextualized joint influence with other psychosocial and sociodemographic factors, kindness fits equally goal- and circumstantial- self-regulation, as well as integrative organization of information. Kindness is a principle that optimizes a refreshing and prosocial interaction with the environment. As it anticipates sharing and cooperation behaviors, it might have a primordial function on individual and social development.

## Introduction

When it comes to developmental education, some of the best parents' wishes for their children would be described as the optimization of their wellbeing, through an autonomic and harmonic interaction with their environment. Autonomy can be described as experiencing life on an active and deliberated way, with a favorable impact in the quality of life and in the accomplishment of social commitments.

On the contrary of adaptive behaviors, which are focused in fulfilling the environmental demands, inferring a passive interaction with the environment, the educators and health carers would encourage autonomous behaviors, that are guided by meaningful purposes, defined individually. In fact, the autonomous behaviors of interest might favor a more integrated society, with reciprocal benefits and transformations. These would be driven by preferences that are inclined to promote harmonious relations with others, called prosocial principles (Eisenberg et al., 1998), which would guide the deliberated choices. Among them, there is kindness, that involves care and non-judgmental understanding toward someone (Neff, 2003). Although the economic literature can refer to kindness as a behavior, which involves some donation in time, money or a gift—a meaning closer to beneficence (Baskerville et al., 2000; Neilson, 2009), at this work kindness is described as a filter that drives the initial approach to others, independently of a consequent behavior that would reflect it or not. Different from empathy, which involves the identification of others' emotional state or condition (Eisenberg et al., 1998), kindness is better characterized as a trait expressed right in the initial contact with others, not only in response to their emotional state/condition. When it comes to kindness as a behavior, it has been associated with increased social approval, and among pre-adolescents, it improves peer acceptance and wellbeing (Layous et al., 2012).

As a prosocial inclination, kindness would increase the possibility of favorable interaction with the environment, with a successful adjustment of one's response in novel or challenging circumstances, taking into account rules or goals. This adjustment ability is commonly referred to as executive functions, dependent on the prefrontal and parietal functioning (Bettcher et al., 2016; Tschentscher and Hauk, 2016; Breukelaar et al., 2017). It is described as an ability to organize a sequence of actions, language or reasoning in order to reach a goal (Fuster, 2008). In neuropsychological literature, executive functions have been constantly referred as adaptive functions. More specifically, cognitive control refers to a regulatory process respect to an action, commonly assessed through tasks of performance monitoring, inhibitory control and cognitive flexibility (Bunge et al., 2002; Power and Petersen, 2013), more dependent on medial frontal areas, specifically: the supplementary motor area (SMA) and pre-SMA (medial BA6), the frontal eye fields (medial BA8), the dorsomedial prefrontal cortex (PFC) (BA9) and the Anterior Cingulate Cortex (ACC) (BA32, BA24, BA25) (Ridderinkhof et al., 2004; Posner et al., 2012; Akkermans et al., 2016; Navarro-Cebrian et al., 2016). Other executive functions are more rule-based abilities of organization of the information, like verbal fluency, and concept formation, more dependent on the dorsolateral prefrontal cortex (i.e., Specht et al., 2009; Porter et al., 2011).

These functions are still under development during late adolescence, with increased performance monitoring in a 2 years interval, between 18 and 20 years-old (Mota et al., 2013). During adolescence, there is a marked improvement in executive functions, like concept formation and planning (Luciana et al., 2009; Kleibeuker et al., 2013). On other functions which include a modulation of one's interaction with the environment, specifically inhibitory control and risk taking, although adolescents reach adult levels, they present a differentiated pattern of frontostriatal activation (Hawes et al., 2017).

The neuropsychological improvements provide a unique index of the developing cognitive processes, as well as indirectly inform about the adequacy of the anatomical and physiological brain maturation. Neurodevelopmental changes during adolescence include a heterochronic and nonlinear reduction of the frontal and parietal gray matter density, as well as a simultaneous, linear and diffuse increment in the white matter (Giedd et al., 1999; Sowell et al., 1999; Snook et al., 2005; Giorgio et al., 2008). These transformations are accompanied by a reorganization of the brain sulci and giri (Blanton et al., 2001).

Remarkedly, some of these neurodevelopmental transformations, like the volume increase of the hippocampal complex and some subcortical structures, still occur at least until early adulthood (Ashtari et al., 2007; Golarai et al., 2007; Lebel et al., 2008). Regarding the physiological transformations - specifically, neurohormonal levels changes, increase of brain blood flow and reduction of glucose consumption—they already reach adult levels during adolescence (Takahashi et al., 1999; Weise et al., 2002). Taken together, these results inform about a pattern of non-linear reorganization that bases the neurodevelopment at late adolescence.

However, less is known about the reorganization of psychological phenomena at this period of life, with scarce understanding of the association between executive functions and psychosocial factors. In fact, this is a developmental period highly influenced by improved social interaction. Adolescents are more prone to look for peers and consolidate relationships based on trust outside the familiar nucleus, updating the quality of their social interactions. Among the prosocial behaviors more mentioned by adolescents in focus groups, there is a highlight for those which favor other's wellbeing and social integration, namely: stand up for others, emotional support, help others develop skills, compliments/encouragement and inclusion (Bergin et al., 2003).

An evolved understanding of commonalities between neurocognition and prosocial inclinations might advance developmental courses through more targeted and integrated techniques in educational and clinical programs. In order to achieve that, the research methodology might resemble more directly the real-world, getting closer to the clinical adopted methods to reach conclusions.

Clinical neuropsychological practice suggests that the influence of factors does not occur isolatedly, but rather in a contextualized way. For example, for an 8 years-old child who does not read, the information about age is not informative itself. When this age occurs in the presence of other factors, like extensive and/or individualized training, its informative composition changes. In other words, one factor behaves in a different way, according to the setting of other factors around. Thus, context is a crucial feature to understand the tessellation that describes the psychological phenomena.

It is important to point out that such contextual interaction is not constraining as in a chemical combination in which the original factors disappear giving place to a resulting new element. Rather, it is influential. It motivates a transformation in the way the factor manifests itself, without an external determination.

This approach is specially relevant in inhomogeneous societies, with high socioeconomic discrepancies, like Brazil (Ferreira et al., 2016). Such influence might be not as noted in more homogeneous societies, considering that some factors might be constant across the population and their influence modulating the psychological phenomena of interest can be ignored.

Indeed, even considering only neurocognitive performance, the same manifestation (e.g., an 8 years-old child who does not read) can reflect different underlying mechanisms (e.g., dyslexia, acquired brain injury, dystaxia). This phenomena can be described as different configurations of the involved/latent factors, resulting in the same manifestation. Therefore, most harmonic manifestation would be achieved with the best manifestation of each involved factor in that configuration of all those elements. Thus, relative values achieve more relevance in the study of psychological phenomena than absolute values.

The classic approach of the involved factors as independent has kept the predictions about psychological clinical establishment and evolution far from accuracy. Traditionally, the statistical techniques which consider the influence of factors over a dependent variable treat those factors as independent (e.g., bivariate correlation, linear regression) or assume their interaction as a new resulting data. It occurs with the partial correlation, in which the independent value is residualized from intervenient variable. Even in multiple regression, the factors included in a proposed model are assumed as absolute values which sum contributes to predict the dependent variable. The stepwise regression has the advantage of considering a variety of models and selecting the more fitting variables for the selected model, however it still considers the contribution of some factors to the other ones as additive.

From a conceptual framework regarding the psychological phenomena, we intent to resemble the contextual relationship in this study design. Thus, the factors will not have the same weight in every model. Each factor parameter in each model will be weighted and valued according to the presence of other parameters. So, instead of absolute values, relative parameters will be used for analyzing kindness and the factors that interfere on its impact over the neuropsychological performance.

Therefore, this study aimed to investigate the following hypotheses: (1) kindness would be related with the executive functions. If so, it would be more associated with measures of self-regulation, mainly dependent on the medial prefrontal corticosubcortical circuits, than with measures of organization of the information, mainly dependent on the dorsolateral prefrontal areas. (2) among self-regulating processes, kindness would be more associated with autonomic responses—choices guided by one's understanding/intention—than with adaptive responses—changes on one's choices triggered by unfavorable circumstances.

## Materials and methods

### Sample

A sample of 46 (31 female; 18 to 21 years-old) healthy college students from different courses of the University of the State of Rio de Janeiro participated in this study. Inclusion criteria, in order to obtain a more developmentally homogeneous sample, included: age up to 21 years-old and enrollment at first or second term. The exclusion criteria included: acquired brain damage or neurological disorder (including loss of consciousness for at least 20 min and developmental disorders); prenatal and perinatal complications (congenital infection, abusive maternal consumption of psychoactive substances, hypoxia); psychopathological disorders (Axes I and II DSM- IV); regular and intensive drug (alcohol, cannabis, opiates, hallucinogens, cocaine, amphetamine compounds or psychoactive substances medically prescribed); and sensory or motor uncorrected difficulties.

### Procedure

Once recruited through signs, emails and class visits, participants attended a 1 h clinical interview. There, the interviewer explained the nature of the study, obtained their informed consent, and collected their clinical and sociodemographic information. At this moment, participants provided information about their university course, years of education, neighborhood, and their parents' educational level and profession. Their clinical history was explored through questions about previous diagnostics, learning difficulties and neurological procedures or psychiatric services. Their current clinical symptomatology was addressed through structured questionnaires and questions about their adaptation in the university as well as about their habits of tobacco, alcohol and psychoactive drugs consumption. Once confirmed their eligibility, they attended a (1:30 h) comprehensive neuropsychological assessment within one or 2 weeks. Both interview and assessment were conducted by trained students of Psychology. This study respected the fundamental principles of human rights stated in the Declaration of Helsinki and in the national legislation about bioethics. Once explained the objectives and procedures of this study, all subjects gave written informed consent. It was approved by the national research ethics committee, named “Comitê Nacional de Ética em Pesquisa”.

### Instruments

The clinical interview included a semi-structured questionnaire; the M.I.N.I. Questionnaires: Major Depressive Episode, Obsessive-Compulsive Disorder, Psychotic Disorders, Generalized Anxiety Disorder, Antisocial Personality Disorder (Lecrubier et al., 1997); as well as the Handedness Edinburgh Inventory (Oldfield, 1971).

The neuropsychological assessment was focused mainly in the executive functions. The instruments presented below were administered in the following order: STROOP, d2, Self-Ordered Pointing Task (SOPT), Wisconsin Card Sorting Test (WCST), Letter Number Sequencing (WAIS-III), Verbal Fluency (Phonetic), Zoo Map (BADS), Vocabulary (WAIS-III), Matrix Reasoning (WAIS-III), and Iowa Gambling Task.

In order to address our hypothesis, the variables were selected according to their fit to the objective. They were classified as: organization of the information, when they represented the selection of rule-matching information; or self-regulation, when they represented a response monitoring or update. These self-regulating variables were sub-classified as autonomic (based on previous own choices) or adaptive (based on environmental circumstances). Following, they are listed according to this classification:

ORGANIZATION OF THE INFORMATION
  Matrix Reasoning (WAIS-III; Wechsler, 2004):
  The participant must select one of four/five options which would complete the showed series of abstract pictures. It measures visual concept formation and is used as an estimation of nonverbal premorbid general functioning. It has been associated with other measures of problem solving and verbal reasoning (Dugbartey et al., 1999) and its association with the prefrontal functioning is not clear (Tranel et al., 2008).
Vocabulary (WAIS-III; Wechsler, 2004):
  The participant must formulate a precise definition of 33 words increasingly difficulty. It values verbal comprehension and concept formation and is used as an estimation of verbal premorbid general functioning (Wechsler, 2004). It presents high indexes of reliability, represented by test-retest and internal consistency superior to 0.90 (Barr, 2003). Its association with the prefrontal functioning is not clear (Tranel et al., 2008).
Verbal Fluency (Phonetic) (Strauss et al., 2006):
  The participant is asked to say, as fast as possible, many words that begin with the letter F, A and S (in 1 min each). Proper names and derivative forms are not accepted. The main processes related to this task are the categorization, processing speed and working memory (Strauss et al., 2006). The phonetic verbal fluency is associated with the activation of posterior regions from the left frontal lobe (Birn et al., 2010; Sanjuán et al., 2010). It shows considerable test - retest correlation (r >0.70) and ecologic validity (Strauss et al., 2006). Its accuracy (total) score has been analyzed in this study as an index of organization of the information. Its errors score has been added as a negative index of goal-driven self-regulation.

GOAL-DRIVEN SELF-REGULATION
  Zoo Map (Behavioral Assessment of Dysexecutive Syndrome; BADS; Wilson et al., 1996):
  This task has two versions. On both, the participant is asked to trace a route in order to visit six (out of twelve possible) places, marked in a zoo map. When planning the route, they must follow some informed rules. In the first version, the participant needs to plan the route. In the second version, the traject is already indicated. This task values planning and is associated with the prefrontal functioning. It shows a test-retest index inferior to 0.70 and its validity index is 0.41 (Wilson et al., 1996; Norris and Tate, 2000).
Letter-Number Sequencing (Wechsler Adult Intelligence Scale; WAIS-III; Wechsler, 2004):
  The participant is read increasing sequences of numbers and letters and recalls the numbers in ascending order and the letters in alphabetical order. It involves working memory and cognitive flexibility. It presents high reliability index (*r* >0.85) and moderate correlation with other executive functions subtests (digit span and arithmetic) (Wechsler, 2004).
STROOP (Stroop, 1935):
  This task has 3 trials (100 stimuli each), in which the participant needs to answer as fast as possible. On the first (Word), the participant is asked to read color names. On the second (Color), they are asked to name colors. On the third (Word Color), a name of one color is printed in the ink of another one. The participant is asked to say the ink color of each word. This task involves conflict monitoring and inhibitory control. It is sensitive to brain injury and is associated with the prefrontal functioning (Guise et al., 2014; Cipolotti et al., 2016). It showed moderate to high reliability coefficients (Franzen et al., 1987) and has ecologic validity (Garcia-Molina et al., 2012).

CIRCUMSTANTIAL-DRIVEN SELF-REGULATION
  Wisconsin Cards Sorting Task (WCST; Grant and Berg, 1948):
  The participant must match each of the 128 cards with one of the four model cards displayed in front of them. After each classification, the examiner says if the answer is correct or not. There are three classification criterias (color, shape and number), which change without previous notification, after 10 consecutive correct matches. The task ends when the sequence color-shape-number is completed twice or when the cards are over. It assesses the concept formation, cognitive flexibility and the capacity to adapt to external contingencies. Its performance is associated with the dorsolateral prefrontal functioning. This task is sensitive to prefrontal injuries, and shows varied test-retest index and consistent ecologic validity (Strauss et al., 2006).
Self-Ordered Pointing Task - abstract figures (SOPT; Petrides and Milner, 1982):
  On this task, the participant is given a stimulus booklet with 108 foils, which show abstract drawings. It has four blocks (with three trials each), with increasing number of stimuli (six, eight, ten, and twelve). The stimuli are repeated in every foil of each trial, but their position is changed on each following foil. The participant is asked to sign a drawing on each foil, without repeating the anteriorly signed ones. This task informs about the ability to regulate the behavior using plans and strategies. It is related with the processing speed and the working memory. It is sensitive to the prefrontal damage and its performance is associated with the everyday life activities (Strauss et al., 2006). It presents varied correlation (*r* = 0.23 a 0.52) with other executive functions measures (Daigneault et al., 1992; Bryan and Luszcz, 2001). The test-retest index oscillates between 0.38 and 0.76 (Shimamura and Jurica, 1994; Archibald and Kerns, 1999).
Iowa Gambling Task (IGT; Bechara, 2007):
  On this computerized task, there are four decks (A, B, C, and D) on the screen. The participant must select cards from any of them. After each selection, the participant wins money and sometimes they also lose money. Their goal is to obtain as much money as possible and they are informed that they are only eligible to win the game if they identify and avoid the worst decks. Also, the computer does not change the decks sequence and does not punish randomly nor according to their previous choice. The task ends after 100 selected cards (Bechara et al., 2000). The decks A and B are disadvantageous because their selection always results in negative net score (high immediate gain and bigger long-term loss). The decks C and D are advantageous; result in positive net score (low immediate gain and lower long-term loss). The decks A and C are similar respect to the high frequency and low magnitude of immediate loss (frequent loss of low quantities). The decks B and D are characterized by low frequency and high magnitude of immediate loss (low frequent loss of high quantities) (Bechara et al., 2000).
  This task was designed to measure the decision making ability, a process related to the ventromedial and orbitomedial prefrontal functioning (Bechara et al., 1999; Bechara and Damasio, 2005; Dunn et al., 2006). Its validity is reflected by its positive relation with working memory and other executive functions (Buelow and Suhr, 2009).

Kindness was measured by the referred subscale from the Compassion Scale (Pommier, 2010). This scale presents moderate content, convergent and discriminant validity, with a reliability of 0.90 (Pommier, 2010). As it is a self-report, the participants might answer according to the society expectancies, what is referred to as social desirability. So, in order to control for its effect on the scores, the Marlowe-Crowne Social Desirability Scale has been employed. Its items list situations of extreme adequation to the society demands. It has high indexes of validity, *r* = 0.90 and reliability, *r* = 0.85 (Ribas et al., 2004).

### Statistical analysis

A generalized non-linear model analysis was performed in R (R Core Team, 2016), a flexible statistical platform, using the package “gnm” (http://go.warwick.ac.uk/gnm). In cases that the predictor was found to be linear, a generalized linear model was performed. Despite our objective was to address the association between kindness and executive function, we included other relevant factors to the models, in order to reach a more contextualized characterization of their association. For each neuropsychological variable, 93 models were constructed with every possible combination among the following factors: kindness, social desirability, gender, age and mother's level of education, graduation course and/or term. In general, these factors have a small number of levels of response, what restricts their possibilities of linearity. As the considered neuropsychological measures at this period of life are on advanced developmental stage, many of them did not reach normality, being the poisson distribution assumed. Therefore, only neuropsychological variables with positive value were used.

**Figure 1:**
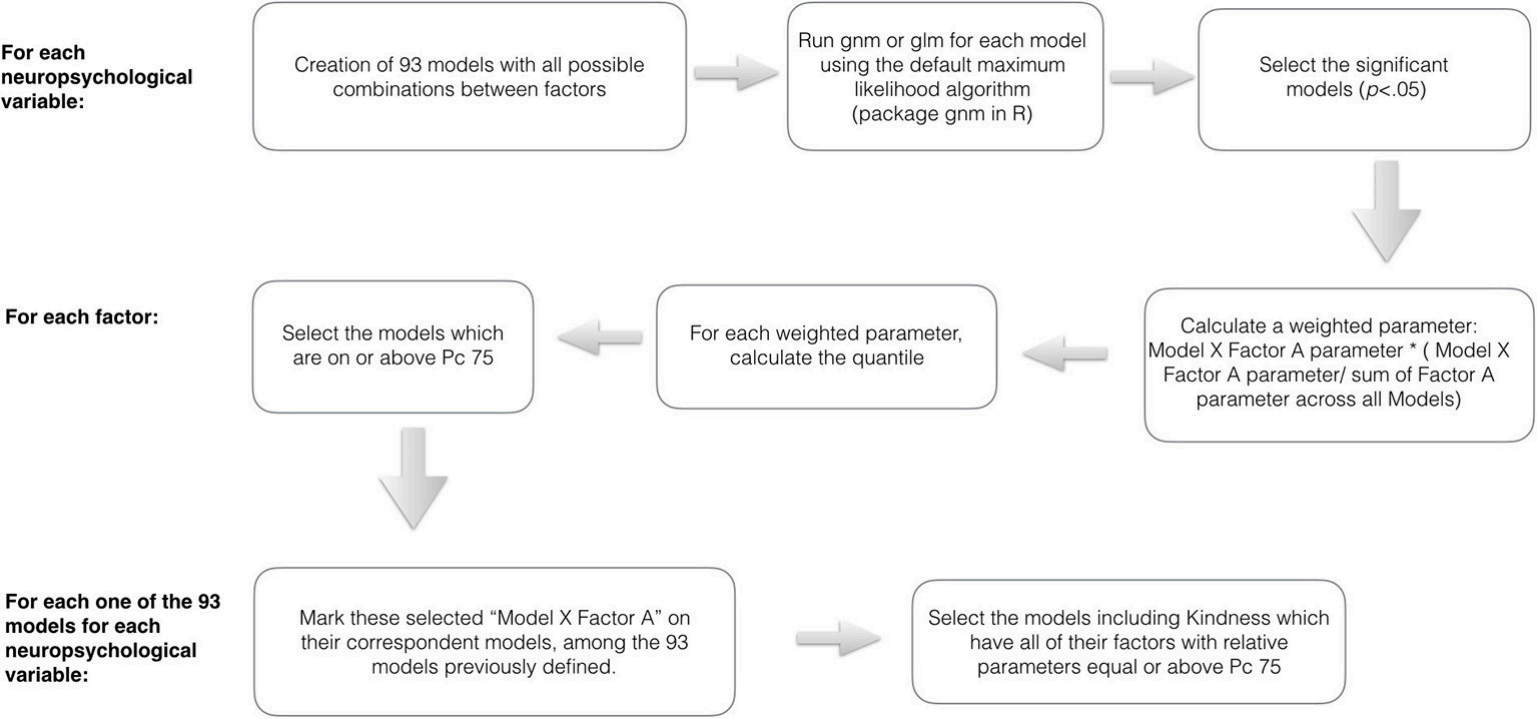
Schematic illustration of the data analysis steps toward the identification of harmonic and contextualized models.

In an attempt to reach the significant models which presented the factors in their maximum expression (weighted parameter across models equal or above 75 quantile), we proceeded to some steps schematically illustrated in the Figure 1.

In order to represent more accurately the professional conclusions, the neuropsychological results were interpreted through a contextualized approach. Driven by the objectives of the assessment, the qualitative analysis of each quantitative variable was made in comparison with the other variables' results, as the whole setting is more informative of the participants' neuropsychological pattern.

## Results

The sample's sociodemographic characteristics are shown in Table 1. The mean score on kindness, social desirability and the neuropsychological tests are shown in Table 2.

**Table 1:** Sociodemographic characteristics of the sample.

**Variables**	***Valid n***	**%**
**AGE**		
18	8	17.4
19	19	41.3
20	14	30.4
21	5	10.9
**GENDER**		
Male	15	32.6
Female	31	67.4
**HANDEDNESS**		
Dexterous	43	93.4
Ambidextrous	3	6.6
**UNDER GRADUATION COURSE**		
Psychology	20	43.5
Nutrition	13	28.3
Nursing	2	4.3
Biology	1	2.2
Laws	1	2.2
Geology	1	2.2
Odontology	2	4.3
Electrical Engineering	4	8.7
Journalism	2	4.4
**CITY**		
Rio de Janeiro	26	56.5
Near cities	20	43.5
**TOBACCO CONSUMPTION**		
Never	46	100
**ALCOHOL CONSUMPTION**		
Never	30	65.2
Occasionally	15	32.6
**MOTHER'S EDUCATIONAL LEVEL**		
Elementary school	17	37.0
High school	20	43.5
Undergraduation or higher	8	17.4
**FATHER'S EDUCATIONAL LEVEL**		
Elementary school	6	13.0
High school	29	63.0
Undergraduation or higher	8	17.4

**Table 2:** Mean and Standard Deviation (SD) for kindness, social desirability and the neuropsychological tests.

**Variables**	**Mean**	**SD**
Kindness (Scale of Compassion)	16.78	3.66
Social Desirability Scale	13.38	5.86
**WAIS-III LETTER-NUMBER SEQUENCING**		
Total	10.75	2.46
Span	5.41	0.87
**SOPT**		
6 pictures (errors)	1.00	1.06
6 pictures (repetitive errors)	0.26	0.54
8 pictures (errors)	1.76	1.87
8 pictures (repetitive errors)	0.21	0.42
10 pictures (errors)	2.26	2.14
10 pictures (repetitive errors)	0.22	0.69
12 pictures (errors)	3.05	2.73
12 pictures (repetitive errors)	0.24	0.49
Total (errors)	8.05	6.80
Total (repetitive errors)	0.88	1.33
**WCST**		
Errors (%)	31.61	17.32
Perseverative errors (%)	16.81	12.87
Perseverative responses (%)	16.97	10.99
Conceptual level responses(%)	58.46	22.36
Completed categories	4.80	1.71
Failure to maintain set	0.88	2.47
Learning to learn	-0.11	12.81
**VERBAL FLUENCY (PHONETIC)**		
F	14.16	4.47
A	12.34	4.79
S	11.98	4.09
Total	38.75	11.92
Errors	0.77	1.60
**BADS ZOO MAP**		
Version 1: sequence score	5.37	2.53
Version 1: errors	1.69	2.05
Version 1 score: sequence score–errors	3.76	3.92
Version 1: planning time (sec)	48.44	62.31
Version 1: execution time (sec)	196.51	77.93
Version 2: sequence score	7.93	0.33
Version 2: errors	0.40	0.86
Version 2 score: sequence score–errors	7.09	0.66
Version 2: planning time (sec)	9.88	16.02
Version 2: execution time (sec)	87.88	37.54
Version 1 + Version 2 scores	11.15	4.14
**STROOP**		
Word	83.07	17.86
Word (errors)	0.12	0.32
Color	67.14	12.75
Color (errors)	0.19	0.55
Word Color	45.37	11.27
Word Color (errors)	0.81	2.92
Interference	11.93	11.50
**IGT**		
Standard score: (A + B)–(C + D)	27.31	24.31
Sensitivity to frequency score: (B + D)–(A + C)	30.63	21.45
Deck A	15.05	6.21
Deck B	30.90	12.64
Deck C	22.78	14.22
Deck D	31.02	13.79
WAIS-III Vocabulary	35.68	10.08
WAIS-III Matrix Reasoning	18.95	4.25

It was hypothesized that kindness would relate with executive functions. This contextualized analysis, which looks only for significant models with all of their factors in their group's top positions, identified that 21 out of the 62 models including kindness were significantly fitting and presented their higher contextual levels (all factors above Pc 75). Indeed, models which presented only kindness as significant predictor were observed among the Organization of Information variables as well as among the Circumstantial-Driven Self-Regulating (see Figures 1, **3**).

**Figure 2:**
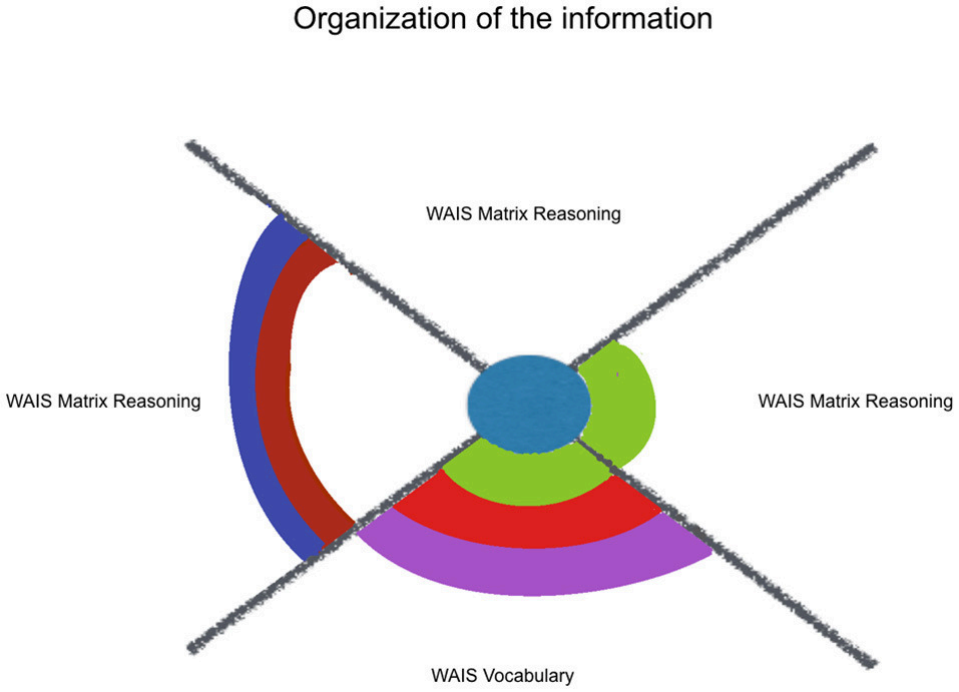
Kindness estimation of neuropsychological performance, considering relative and joint influence of psychosocial and sociodemographic factors: organization of information. Legend: Significant (non-)linear models with all of their factors weighted above Pc 75 (among all the models including each of them). Kindness in blue, Social Desirability in green, Mother's Educational Level in red, Sex in purple, Course in brown, Age in lilac.

**Figure 3:**
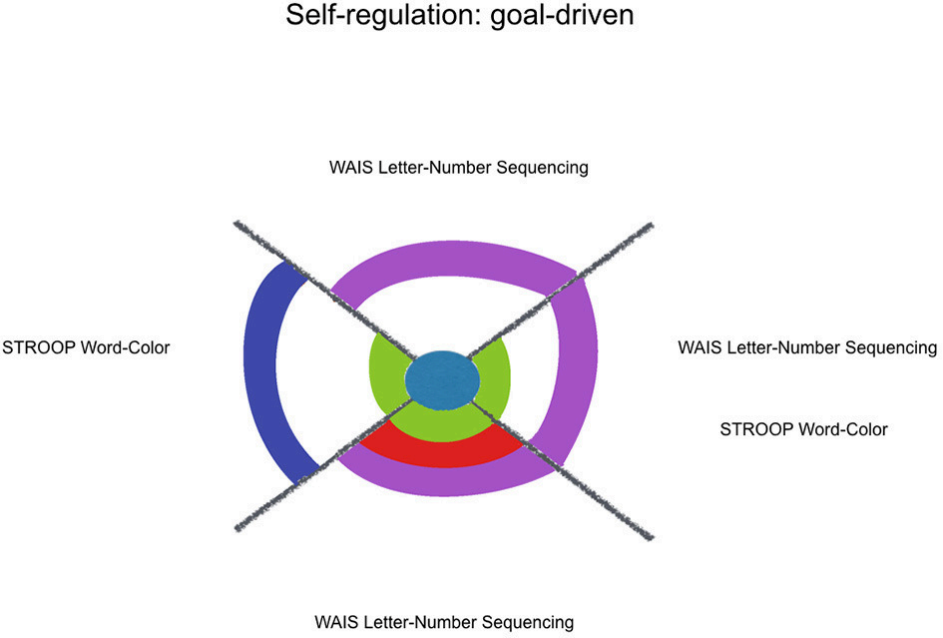
Kindness estimation of neuropsychological performance, considering relative and joint influence of psychosocial and sociodemographic factors: goal-driven self-regulation. Legend: Significant (non-)linear models with all of their factors weighted above Pc 75 (among all the models including each of them). Kindness in blue, Social Desirability in green, Mother's Educational Level in red, Sex in purple, Age in lilac.

Additionally, it was hypothesized that kindness would fit more neuropsychological measures that index monitoring and updating one's own behavior/choices (mainly mesial functions), than with variables that reflect organization of the information (mainly dorsolateral functions). This hypothesis was not supported by our results, that showed the same ratio of 1.3 models/variables for self-regulating indexes and for organizing indexes (see Figures 2, 3). Kindness was as effective to fit executive functions regarding the regulation of one's responses as well as regarding the organization of information. However, respect to this last sub-classification, those variables that were estimated by kindness, WAIS-III Vocabulary and Matrix Reasoning, differ from the not estimated variable, Verbal Fluency (Phonetic) total. The former ones rely on the integration of environmental and own information, through a formulation of an idea, more than listing information, through classification or categorization. Indeed, among the other considered factors, social desirability exerts a joint influence in the estimation provided by kindness for both WAIS-III Vocabulary and Matrix Reasoning. So, those variables, which are also treated in literature as indexes of general verbal and non-verbal intelligence are related with concerns about the community. These integrative cognitive processes might benefit from kindness, as well as the self-regulating ones, even in a non-cognitive targeting intervention.

**Figure 4:**
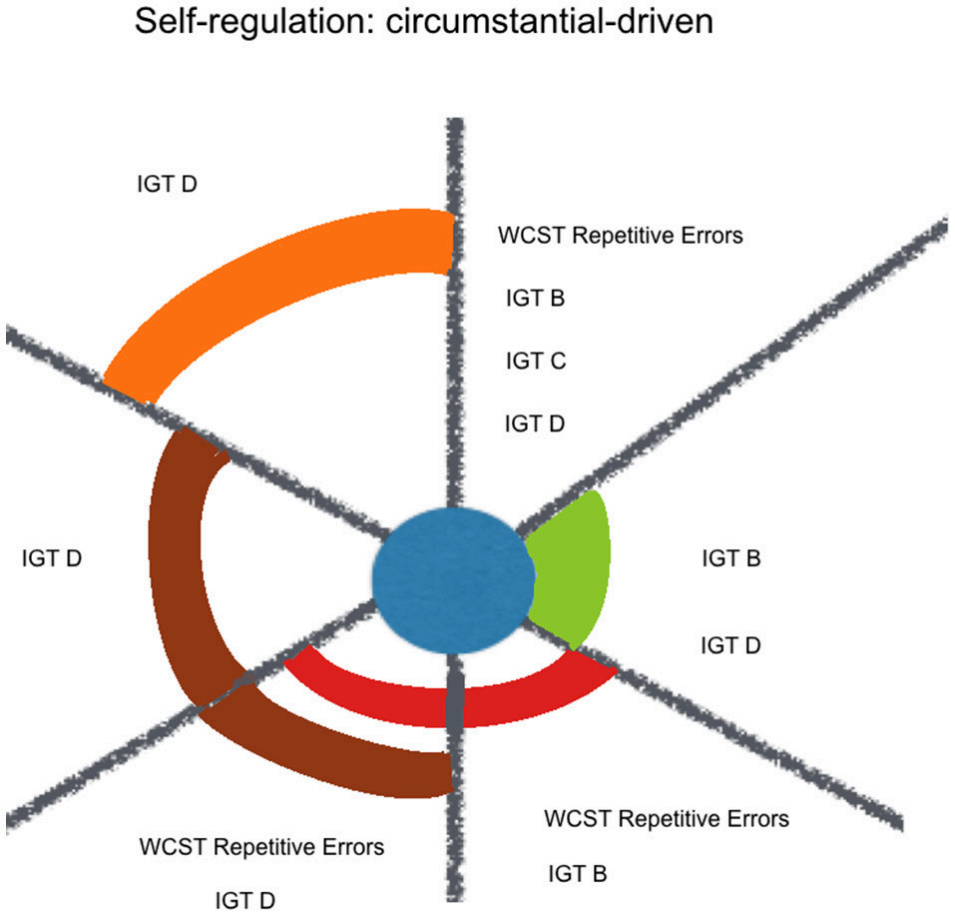
Kindness estimation of neuropsychological performance, considering relative and joint influence of psychosocial and sociodemographic factors: circumstantial-driven self-regulation. Legend: Significant (non-)linear models with all of their factors weighted above Pc 75 (among all the models including each of them). Kindness in blue, Social Desirability in green, Mother's Educational Level in red, Sex in purple, Course in brown, Term in orange.

A contextualized analysis of the results suggests that regarding goal-driven variables, kindness fitted some externally-defined goals (WAIS-III Numbers and Letters Sequencing and STROOP), more than a response update based on one's previous choices (WAIS-III Numbers & Letters Sequencing, and Verbal Fluency Phonetic errors). When it comes to circumstantial-driven self-regulation, kindness fits response update variables that indicate an adjustment mechanism to new environmental circumstances, with feedback (WCST perseverative errors, IGT B, IGT C, IGT D) but not without feedback (SOPT [12 pictures] repetitive errors) or under high magnitude of both gain and loss (IGT A) (see Figure 4).

Among the other considered factors, social desirability and sex were constant factors with joint influence over the estimation of Goal-Driven Self-Regulating variables. It might reflect a female preference for externally-defined goals, like the expected fixed answers for the items in WAIS-III Numbers and Letters Sequencing and STROOP. In the other hand, mother's educational level was highly expressive among the Circumstantial-Driven Self-Regulating variables, what highlights modeling as an important contributor for culturally developed inclinations.

## Discussion

### Kindness as an index of self-regulation

Kindness, described herein as a care and tender inclination toward others on unfavorable circumstances, seems to be an index of self-regulation, as it estimates performance monitoring.

The cognitive processes measured by these variables are not dependent on the consequences, and express the participant's own monitoring, respect to their goals and previous responses. They have been associated with the activation of the medial frontal areas (Ridderinkhof et al., 2004; Posner et al., 2012; Akkermans et al., 2016; Navarro-Cebrian et al., 2016). When the person detects an error, as measured by the Error-Related Negativity (ERN), or adjusts their response according to the environment, as indexed by the feedback-related negativity (FRN), these regions are activated more prominently and they increase the attentional resources allocated for the performance (Li et al., 2008; Motomura et al., 2015; Navarro-Cebrian et al., 2016). The goal-driven error detection might also engage other adjustment mechanisms, like the attribution of more/less relevance (relative value) or valence (value) to the task performance, resulting in improvement or quitting. Also, improved monitoring can activate differentially these regions even without error detection, on a pre-response conflict, due to stimuli complexity (Carter et al., 1998; Donkers and Van Boxtel, 2004) or on an purposeful optimization of the response, like motivation (Maruo et al., 2016) and meditation (Fox et al., 2016). Likewise, kindness seems to improve the relevance or valence of the task rules and optimizes the performance monitoring. In fact, sensitivity to aspects of care has been associated with greater activation of performance monitoring neural basis, the ventral posterior cingulate cortex (Robertson et al., 2007). Therefore, kindness, as well as goal-driven performance monitoring, might not be described as reactive, but as an active approach that occurs at the right contact with the processed information. When the neural basis of performance monitoring are dysfunctional, kindness might act as a moderator improving their functionality on everyday life. Future neuroimaging and neuropsychological studies would clarify its value for neurorehabilitation.

As monitoring response is still developing on late adolescence (Mota et al., 2013), as well as its neural bases (Ashtari et al., 2007; Lebel et al., 2008; Ordaz et al., 2013), it remains to be elucidated if monitoring response is the most complex cognitive correlate of kindness and if this relation is differential across genders.

Kindness also estimated variables regarding decision making under variable magnitude and frequency of gain and loss. These variables integrate the Iowa Gambling Task, which has been associated with the ventromedial and orbitoventral prefrontal cortex functioning (Bechara et al., 1999, 2000). According to behavioral analysis, these areas develop in a different trajectory, compared with the dorsolateral prefrontal cortex (Hooper et al., 2004). Although some studies refer to those regions' functionality as value-based decision, they consider that the value is determined by external contingencies, from an adaptive approach (i.e., Pujara et al., 2015). In the other hand, some recent studies have remarked their association with values defined by the own person, previously or at the contact with the contingencies - like those reflected by optimism (Dolcos et al., 2015), defined as the inclination for favorable expectancies about the future (Carver et al., 2010).

Kindness also interacted with some indexes of organization of the information. These expressed a more autonomic process of integration of the environmental information, resulting in a formulated visual concept or verbal description. This creative mechanism seems to mature still in the adolescence (Kleibeuker et al., 2013) and its interaction with kindness might be an index of cognitive and social development.

### Kindness-related self-regulation might occur through autonomic mechanisms

Our results suggest that kindness fits both self-regulating processes, kindness might be associated with autonomic responses—choices guided by one's understanding/intention—and adaptive responses—one's choices triggered by changes in circumstances.

Some studies refer to kindness and to executive functions as context-dependent processes. However, they describe kindness as the behavior itself (i.e., Baskerville et al., 2000; Neilson, 2009) and executive functions as an adaptive ability (i.e. Barkley, 2001; Dishion, 2016), due to the update on one's response after invalidating feedback. Another possible interpretation of this adjustment mechanism would consider the external cue as a validation measure, more than a reference measure. So, taking into account the external cue, the person changes their response. However, the change occurs in order to keep their own goal or criteria. Some advances on speech processing literature resemble this perspective. Subtle changes on the voice feedback, provoked by pitch perturbation, make the person change their voice production in a direction so they can keep listening to their reference value (Houde and Jordan, 1998). So, the environmental changes stimulate a response update in order to fit the person's model. This is an autonomic adjustment that might occur without deliberation, as many of the participants are not conscious of the voice feedback changes. When it comes to deliberate adjustments on the interaction with the environment, as measured by the executive functions, it is feasible that the update of one's responses indicate that they are guided by a representation (of a value), more than by a model repetition, which would incline them for a fixed response. The attribution of importance is noted when the environmental reference does not meet one's reference and it might be, at least partially, determinant of the transition between the reward-based—mediated by the striatal functioning (Hori et al., 2009; Kim et al., 2009)—and the inclination-based decisions—mediated by medial cortices (Dolcos et al., 2015). Both can be updated with context changes, however at the first, the context is the determining reference, whereas on the later, the context is the validation reference. Among monkeys, the disruption of a medial cortex, the anterior cingulate cortex, affects the behavioral adjustment in a way that the feedback is not relevant for it (Kennerley et al., 2006). Therefore, the mechanisms of adjustment to the environment seem not to be only feedback-based and kindness is more associated with the self-referred ones.

Neurodevelopmentally, these medial frontal regions present a more specialized functioning during the adolescence, with the establishment of segregated functional circuits involving the medial prefrontal cortex and the cingulate-opercular regions (Galvan et al., 2006; Fair et al., 2007). With reinforcement, the ventral striate presents more activation at middle adolescence (14–15 years-old), meanwhile, without reinforcement, the orbitofrontal is still less activated than among young adults (Van Leijenhorst et al., 2010). So, as a step further from the attribution of value, mediated by the contingencies, the attribution of relevance (scale of values) might be a developmental mechanism that underlies the specialization of medial frontal cortices in the autonomic interaction with the environment. Kindness might reflect the attribution of importance to harmony and might favor self-referred value-based choices.

Therefore, our results suggest that kindness is an inclination that promotes the adjustment of one's response in order to keep their reference (values or goal) integrated with the circumstances, contributing to an improved neuropsychological performance, specially on novel or unpredicted situations, that require executive functioning.

### Kindness analysis suggests that accuracy is not the opposite of errors

Although there is an implicit understanding in literature that error answers are antonym of correct answers, our results suggest that they might express processes that are differentially modulated by kindness (as in Verbal Fluency and STROOP).

Accuracy seems to inform about capacities, meanwhile errors seem to be more elucidative of differential dysfunctional or optimizing mechanisms. These differences have been observed among participants with neuropsychiatric disorders, reflecting error processing differences on the flexible adjustment to a changing environment (Manoach et al., 2016). Kindness and some other principles might be reflected by this differential pattern. How someone is inclined toward the situations might be partially determinant for the task performance: not affecting the skills, but providing some level of variability, with more or less optimization of them. More theoretically-based refined designs and complementary techniques, like the magnetoencephalography (MEG), would help to elucidate the psychosocial processes reflected by the response patterns.

## Limitations and further considerations

As a pioneer study, many questions might be arisen, as in which extent do these results represent principles in general, prosocial inclinations, or kindness specificities. Studies about social factors have some intrinsic limitations, as cultural differences and self-reports. Deeper description about the participants' kindness would contribute to further comparison across cultures and studies.

There remains to be elucidated if kindness is age-dependent and its interaction with the executive functions developmental trajectory. If more kindness during childhood would improve executive function on late adolescence, when the strategies —use of representations—favor the performance (Sowell et al., 2001; Beebe et al., 2004; Conklin et al., 2007), educational policies would benefit from incorporating prosocial attitudes among their objectives and methodologies. Initiatives have stimulated kindness (as behavior) in the educational environment, as the Random Acts of Kindness (Dupree, 1996, cited by Baskerville et al., 2000).

In our current western social organization, the relevance given to efficacy requires fixed predicted results, through the automaticity of some equipments and procedures. However, the relevance attributed to prosocial inclinations, like kindness, would increase problem-solving, with more adjusted responses under unpredictable situations. Replication should be changed by spontaneity, favoring the adaptation that makes possible the manifestation of the prior relative importance (values) at the present circumstances - here and now, for Gestalt (Koffka, 2013/1935) or mindfulness (Shapiro and Schwartz, 2000; Shonin et al., 2015).

In general lines, our results highlight the importance of psychosocial aspects as moderators of the neurocognitive functioning. The comprehension of their mechanisms of co-activation and co-development, joint to the neurological functioning, will make it possible to formulate integrated diagnostic and training instruments, more coherent with the everyday life. These instruments would enhance the quality of the neuropsychological services, not only as a tool for differential diagnosis and prognosis in broad mental health, but mainly as a valuable resource to enhance social integration, through rehabilitation or educational methodology.

Kindness is a prosocial principle that optimizes an updated interaction with the environment through mechanisms of integration. It anticipates sharing and cooperation behaviors, which might have a primordial role on individual and social development. It is not reactive, but active, associated with one's own regulation during the moment of the information processing and the response. There seems to be a tune- (principles) based adjustment that modulates the choices and changes the context, challenging its dynamics and one's own development, favoring relational well-being.

## Author contributions

NM designed the work, analyzed/interpreted the data and wrote the manuscript. EC, MA, RB, AP, and VS contributed with the acquisition and analysis of the data, as well as revised and approved the manuscript.

### Conflict of interest statement

The authors declare that the research was conducted in the absence of any commercial or financial relationships that could be construed as a potential conflict of interest.
